# A Saline Intra-venous Injection for the Treatment of Cholera

**Published:** 1885-11

**Authors:** 


### A Saline Intra-venous Injection for the Treatment of Cholera.

M. Hayem ("Pevue Scientifique;" "Lyon Medical) thinks that besides the need of restoring the salts lost from the blood of cholera patients, it is desirable to overcome its acidity, and for that reason he advises the addition of soda to the saline injection employed, as in the following formula:

| | | |
|---|---|---|
| Water | 1,000 | grammes. |
| Chloride of sodium | 5 | " |
| Hydrate of sodium | 1 | " |
| Sulphate of sodium | 25 | " |

The weeping willow seems to have had a romantic history. The first scion, it is said, was sent from Smyrna in a box of figs to Alexander Pope. General Clinton brought a shoot from Pope's tree to America, in the time of the Revolution, which, passing into the hands of John Parke Curtis, was planted on his estate in Virginia, thus becoming the progenitor of the weeping willow in America.—*The Garden.*

